# Interaction between genetics and the adherence to the Mediterranean diet: the risk for age-related macular degeneration. Coimbra Eye Study Report 8

**DOI:** 10.1186/s40662-023-00355-0

**Published:** 2023-08-14

**Authors:** Patrícia Barreto, Cláudia Farinha, Rita Coimbra, Maria Luz Cachulo, Joana Barbosa Melo, Yara Lechanteur, Carel B. Hoyng, José Cunha-Vaz, Rufino Silva

**Affiliations:** 1 Association for Innovation and Biomedical Research on Light and Image (AIBILI), Coimbra, Portugal; 2 Univ Coimbra, Coimbra Institute for Clinical and Biomedical Research (iCBR), Faculty of Medicine, Coimbra, Portugal; 3 Univ Coimbra, Centre for Innovative Biomedicine and Biotechnology (CIBB), Coimbra, Portugal; 4 Ophthalmology Department, Centro Hospitalar e Universitário de Coimbra (CHUC), Coimbra, Portugal; 5 Clinical Academic Center of Coimbra (CACC), Faculty of Medicine, University of Coimbra, Coimbra, Portugal; 6 Cytogenetics and Genomics Laboratory, Clinical Academic Center of Coimbra (CACC), Faculty of Medicine, University of Coimbra, Coimbra, Portugal; 7 Univ Coimbra, Center of Investigation in Environment, Genetics and Oncobiology (CIMAGO), Faculty of Medicine, Coimbra, Portugal; 8 Department of Ophthalmology, Donders Institute for Brain, Cognition and Behaviour, Radboud University Medical Center, Nijmegen, The Netherlands

**Keywords:** Age-related macular degeneration, Mediterranean diet, Genetics

## Abstract

**Background:**

Age-related macular degeneration (AMD) is a multifactorial degenerative disease of the macula. Different factors, environmental, genetic and lifestyle, contribute to its onset and progression. However, how they interconnect to promote the disease, or its progression, is still unclear. With this work, we aim to assess the interaction of the genetic risk for AMD and the adherence to the Mediterranean diet in the Coimbra Eye Study.

**Methods:**

Enrolled subjects (n = 612) underwent ophthalmological exams and answered a food questionnaire. Adherence to the Mediterranean diet was assessed with mediSCORE. An overall value was calculated for each participant, ranging from 0 to 9, using the sum of 9 food groups, and a cut off value of ≥ 6 was considered high adherence. Rotterdam Classification was used for grading. Participants’ genotyping was performed in collaboration with The European Eye Epidemiology Consortium. The genetic risk score (GRS) was calculated for each participant considering the number of alleles at each variant and their effect size. Interaction was assessed with additive and multiplicative models, adjusted for age, sex, physical exercise, and smoking.

**Results:**

The AMD risk was reduced by 60% in subjects with high adherence to the Mediterranean diet compared to subjects with low adherence to the Mediterranean diet. Combined effects of having low adherence to the Mediterranean diet and high GRS led to almost a 5-fold increase in the risk for AMD, compared to low GRS and high adherence to the Mediterranean diet. The multiplicative scale suggested a multiplicative interaction, although not statistically significant [odds ratio (OR) = 1.111, 95% CI 0.346–3.569, *P* = 0.859]. The additive model showed a causal positive effect of the interaction of GRS and adherence to the Mediterranean diet: relative excess risk due to interaction (RERI) = 150.9%, (95% CI: − 0.414 to 3.432, *P* = 0.062), attributable proportion due to interaction (AP) = 0.326 (95% CI: − 0.074 to 0.726, *P* = 0.055) and synergy index (SI) = 1.713 (95% CI: 0.098–3.329, *P* = 0.019). High GRS people benefited from adhering to the Mediterranean diet with a 60% risk reduction. For low-GRS subjects, a risk reduction was also seen, but not significantly.

**Conclusions:**

Genetics and Mediterranean diet interact to protect against AMD, proving there is an interplay between genetics and environmental factors.

**Trial registration:**

The AMD Incidence (NCT02748824) and Lifestyle and Food Habits Questionnaire in the Portuguese Population Aged 55 or More (NCT01715870) studies are registered at www.clinicaltrials.gov. Five-year Incidence of Age-related Macular Degeneration in the Central Region of Portugal (AMD IncidencePT); NCT02748824: date of registration: 22/04/16. Lifestyle and Food Habits Questionnaire in the Portuguese Population Aged 55 or More; NCT01715870: date of registration: 29/10/12.

**Supplementary Information:**

The online version contains supplementary material available at 10.1186/s40662-023-00355-0.

## Background

Age-related macular degeneration (AMD) is a neurodegenerative disease of the macula, responsible for irreversible vision loss in people aged 55 or older [1].The worldwide prevalence of AMD has been estimated to be 8.69% but with longer life expectancies, and an increasing proportion of aged individuals in the population of many countries, AMD prevalence is expected to increase to 288 million people by 2040 [2].

Genetics, age, and smoking history are well-accepted and major risk factors, but obesity, hypertension, dietary fat intake and sunlight exposure have inconsistently been associated with the disease [3, 4]. Evidence has highlighted that the Mediterranean diet may be protective for the development or progression of AMD [5–11], consistent with its role in the prevention of cardiovascular [12, 13], neurological [14] and oncological diseases [15–17].

As AMD is a multifactorial disease, an approach to untangle the pathophysiology is to assess the interplay of different risk factors. Genetics and the Mediterranean diet assume particular interest on this matter, as a study recently evidenced. A population in Sardinia, Italy, showed, despite its longevity, a low AMD prevalence. This population traditionally adhered to the Mediterranean diet, presented a low genetic risk for AMD and disclosed a healthy lifestyle [18].

The genetic background has well been established [19–21] and is unmodifiable. Different genes have been associated with AMD, like genes from the complement system, *ARMS2/HTRA1*, *ABCA1*, *LIPC*, amongst others. Adhering to a healthy diet, however, is an option that may be taken by each patient, and that may allow for a change in the genetic expression and the disease course.

There is scarce and conflicting information regarding the interaction of the genetic risk for AMD and the adherence of the Mediterranean diet. While some showed no association between the genetic profile and the adherence to the Mediterranean diet, others showed that a good adherence was beneficial for either high-genetic risk profiles, low-genetic risk profiles or just related with specific single nucleotide polymorphisms (SNPs) [7–10, 18]. Furthermore, it is not yet known whether the interaction between the Mediterranean diet and genetics, when present, is on an additive or a multiplicative scale.

The objective of this work was to assess the interaction of the genetic risk and the adherence to the Mediterranean diet in a well characterized Portuguese population, in the Coimbra Eye Study (CES).

## Methods

### The Coimbra Eye Study

The CES comprises the Epidemiological Study (NCT01298674, n = 2975 for Mira cohort; n = 3021 for Lousã cohort), the AMD Incidence Study (n = 1617 for Mira cohort) and the Lifestyle and Food Habits Questionnaire in the Portuguese Population aged 55 or more (n = 985 for Mira cohort; n = 1007 for Lousã cohort).

All studies were cross-sectional and population based. The Epidemiological Study determined the prevalence of AMD in two different populations: Mira (coastal) and Lousã (inland). In the the Epidemiological Study, participants were recruited from the Primary Healthcare Units of Mira and Lousã, provided they were at least 55 years old, sex- and age-matched between the two cohorts. The AMD Incidence Study determined the 6.5-year incidence in Mira. The Lifestyle and Food Habits Questionnaire in the Portuguese Population aged 55 or more study was performed at the same time as the AMD Incidence Study and assessed nutrition and behavioural habits of the two populations (Mira and Lousã). Both studies ran between 2016 and 2017. Details of the studies can be found elsewhere [5, 6, 22–24].

Patients signed the informed consent after explanation of study procedures and possible consequences. The studies procedures complied with the tenets of the Declaration of Helsinki and International Conference on Harmonization–Good Clinical Practice Guidelines. The studies obtained the Association for Innovation and Biomedical Research on Light and Image (AIBILI)’s Ethics Committee approval.

### Procedures

This manuscript refers to the data acquired in the AMD Incidence Study (NCT027048824) and Lifestyle and Food Habits Questionnaire in the Portuguese Population Aged 55 or More (NCT01715870).

Shortly, subjects who participated in the AMD Incidence Study went through an ophthalmological assessment by an ophthalmologist, best-corrected visual acuity with Early Treatment Diabetic Retinopathy Study (ETDRS) charts, color fundus photography (CFP, Topcon® fundus camera, TRC-NW8; Topcon Corp., Tokyo, Japan), spectral-domain optical coherence tomography (SD-OCT), fundus autofluorescence (FAF), and infra-red (IR) imaging (Spectralis HRA + OCT Heidelberg Engineering, Heidelberg, Germany). Additionally, a trained study nurse collected data on clinical history and took blood samples from participants who consented for genetic analysis (n = 948 genotyped).

### Lifestyle and food questionnaire

In the Lifestyle and Food Habits Study in the Portuguese Population aged 55 or more, participants answered a validated lifestyle and food questionnaire. In the lifestyle questionnaire, data on demographics, weight, height and waist circumference, education, smoking habits, physical activity (any type of physical activity performed, regardless the duration), and cardiovascular comorbidities were recorded.

The food questionnaire, validated for the Portuguese population [25], comprised 86 food items arranged by eight groups: (1) Dairies; (2) Eggs, meat, and fish; (3) Oils and fats; (4) Bread and cereals; (5) Sweets and pastries; (6) Legumes; (7) Fruits and (8) Beverages and miscellaneous. For each of the 86 food items, participants answered what the frequency of intake was in the last year (never or less than 1 time/month, 1–3 times/month, 1 time/week, 2–4 times/week, 5–6 times/week, 1 time/day, 2–3 times/day, 4–5 times/day, 6 or more times/day), as well as the average serving size (cups, plate, units, mL, g, number of items, tablespoons, teaspoons) and whether the food item was seasonal.

Each food item’s intake was calculated as the average daily consumption, adjusted for the serving size, in grams. In case of seasonal intake, a value of 0.25 was considered to weight for a period of three months.

### Assessment of adherence to the Mediterranean diet (MediSCORE)

In order to assess the level of mediSCORE [26]. Food items from the food questionnaire were grouped into nine groups: vegetables, legumes, fruits, cereals, fish, meat, dairies, alcohol, and a ratio of monounsaturated lipids (mainly olive oil) to saturated lipids (fats). Each of these groups takes up a value of 0 or 1, compared with the median of sex-specific food item consumption (in grams) of the Mira cohort population calculated in the food questionnaire. For healthy groups (vegetables, legumes, fruits, cereals, and fish), consumption above the median was assigned the value 1. For the food groups considered harmful (meat and dairies), consumption above the median was assigned the value 0. Specifically, for alcohol, a value of 1 was attributed to consumptions between 10 and 50 g/day for men and between 5 and 25 g/day for women, as it was considered healthy. Also, for fat, a ratio of monounsaturated/saturated lipids was considered beneficial when above the sex-specific median consumption and, hence, assigned the value of 1 [26, 27]. The quantity of monounsaturated and saturated lipids that each food item contains is given by “The Portuguese Food Composition Database”, which is the reference document for the composition of food item in Portugal.

Additional file 1: Table S1 presents the cut-off values used to assess a beneficial or detrimental consumption of each food group of the mediSCORE.

An overall value was calculated for each participant, ranging from 0 to 9, using the sum of each nine food groups.

**Fig. 1:**
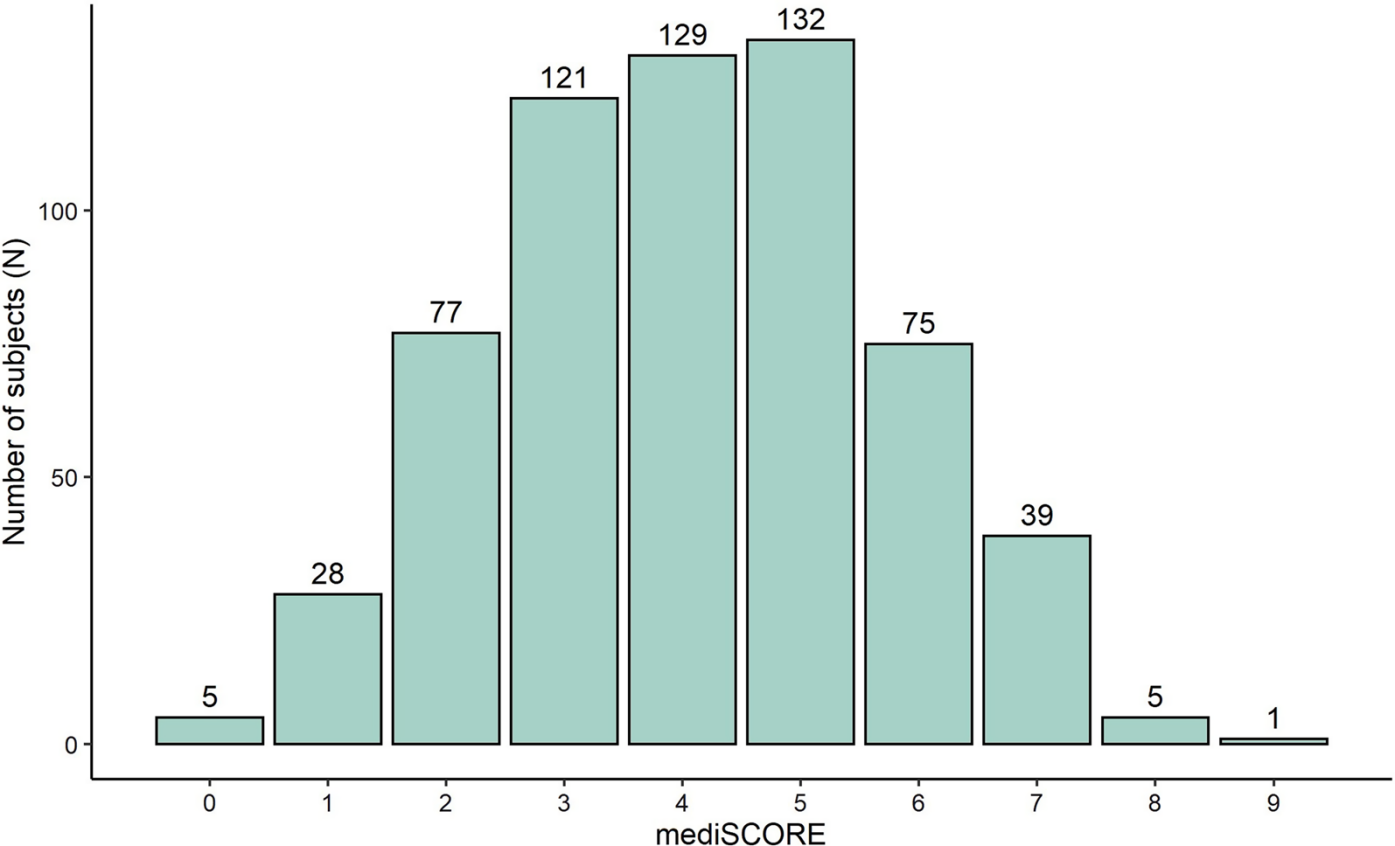
Distribution of the Mediterranean diet score (mediSCORE) in the Mira cohort (n = 612). mediSCORE, adherence to the Mediterranean diet score

High adherence to the Mediterranean diet was expressed by a mediSCORE ≥ 6, which is close to the third tercile of the distribution of the mediSCORE in our sample (Fig. 1). Low adherence to the Mediterranean diet was defined by mediSCORE < 6.

### AMD grading

A general grading (disease/no disease) and a detailed grading (AMD staging) were performed. In cases of bilateral disease, the AMD phenotype was determined by the grading of the most severe eye. In case of only one presenting the disease, this eye was considered for grading. Grading was performed at Coimbra Ophthalmology Reading Center (CORC, AIBILI, Portugal), by certified ophthalmologists. When available, the grading of the two eyes was considered for this analysis.

### Definition of cases and controls and study subjects

Rotterdam Classification was used [28, 29]. Cases were defined as participants with stage 2a (soft, indistinct or reticular drusen), stage 2b (soft, distinct drusen with pigmentary irregularities), stage 3 (soft, indistinct drusen with pigmentary irregularities) and stage 4 geographic atrophy (GA) or choroidal neovascularization (CNV). Controls were participants with stage 0 (no AMD or drusen < 63 μm) above 60 years old, and stage 1 (soft, distinct drusen or pigmentary irregularities) above 70 years old, in order to minimize the chance of classifying participants who could still progress to AMD as controls.

### Genotyping

Genotyping of patients was performed in collaboration with the The European Eye Epidemiology Consortium (E3), within the EYE-RISK project. Detailed information can be found elsewhere [30]. In brief, 69 SNPs described by the International AMD Genomics Consortium [19] were successfully genotyped. Genotyping was performed using single-molecule molecular inversion probes and next generation sequencing to target SNPs and coding and splice-site regions of 10 AMD-related genes (*ARMS2, C3, C9, CD46, CFB, CFH, CFI, HTRA1, TIMP3 and SLC16A8)* and three genes associated with inherited macular dystrophies (*ABCA4, CTNNA1*, and *PRPH2)*. Ten SNPs were genotyped by Kompetitive allele-specific polymerase chain reaction (KASP) genotyping assays to ensure a full genotyping of the 52 variants identified by the International AMD Genomics Consortium (IAMDGC).

### GRS

The GRS was calculated for each participant as the sum of the number of risk alleles ${N}_{i}$, at each variant, multiplied by the risk allele effect size ${\beta }_{i} (log odds-ratio)$from the genome-wide association studies (GWAS) of the IAMDGC fully conditioned analysis, according to the formula $GRS={\sum }_{i=1}^{52}\left({N}_{i}{{\upbeta }}_{i}\right)$.

The 52 variants identified by Fritsche et al. [19] used to calculate the GRS are listed in Additional file 2: Table S2, along with the odds ratios (OR).

Missing genotypes were not replaced by substitute values, meaning no genotype data imputation was performed. Only subjects with all the major risk variants genotyped (*CFH* rs570618, *CFH* rs10922109, *C2/CFB/SKIV2L* rs429608, *ARMS2/HTRA1* rs3750846 and *C3* rs2230199) [19] were considered for the GRS computation. In the absence of at least one major risk variant, GRS was considered null, and the subject was excluded from this analysis. A high GRS was considered equal or superior to the median GRS of the population.

### Statistical analysis

General (demographic, environmental, lifestyle, diet and genetic) characteristics between cases and controls were compared using Mann-Whitney U test and Pearson’s chi-squared test (or Fisher’s exact test) for continuous and categorical variables, respectively. Categorical variables were presented with absolute frequency and percentage, while continuous variables were presented with mean and standard deviation.

The associations of AMD with the mediSCORE and the GRS were estimated with individual eye as the unit of analysis, using a logistic regression model with generalized estimating equations accounting for inter-eye correlation and adjusted for other risk factors, such as age (60–70 years, 70–75 years, > 75 years), sex, smoking (non-smokers, smokers/ex-smokers) and physical exercise. An exchangeable correlation structure was used.

Next, we investigated the interaction between the mediSCORE and the GRS on AMD, to find if the effect of one of the risk factors is different across strata of the other factor. We evaluated the interactions between these two risk factors on a multiplicative and an additive scale. It is recommended to address both multiplicative and additive interactions, although evidence of additive interaction is considered more clinically relevant in epidemiological studies than multiplicative.

We used multiplicative interactions to find if the GRS may multiply the magnitude of the risk to AMD associated with the adherence to the Mediterranean diet. In other words, to see if the effect of the combination of the two risk factors is greater than the multiplication of their individual effects. To do so, a multiplicative term of the two risk factors was added into the logistic regression model described above, adjusted for covariates, and considering the correlation between eyes. Deviations from the multiplicative interaction were tested based on the Wald test statistics.

The other strategy was to compute additive measures to determine whether the combined effect of genetic risk and adherence to Mediterranean diet was more than the sum of the risk of each individually. We computed the relative excess risk due to interaction (RERI), and also two more additive measures for completeness and consistency: the attributable proportion due to interaction (AP) and synergy index (SI). RERI is the part of the total effect that is due to interaction and can be calculated as the difference between the risk when both factors are present and the sum of the risks for each of the two factors (RERI = OR_11_ − OR_01_ − OR_10_ + 1), where OR_11_ refers to the exposure to both factors and OR_01_ and OR_10_, to the exposure to only one of them. AP can be interpreted as the proportion of effect that is due to interaction among subjects exposed to both factors: AP = RERI/OR_11_ and SI is the ratio between combined and individual effect (OR_11_ − 1)/[(OR_01_ − 1)+(OR_10_ − 1)].

Following Knol and Vanderweele recommendations for reporting analyses of effect modification/interaction [31, 32], an improvement on earlier proposals as Strengthening the Reporting of Observational Study (STOBE) was made, and besides the additive and multiplicative measures, we also presented the ORs for the adherence to the Mediterranean diet within strata of the GRS. Additive measures were developed for risk factors rather than preventive factors, so we recoded the mediSCORE protective factor so that the stratum with the lowest risk became the single reference category, when both factors are considered jointly [31, 32]. Therefore, the joint reference category reflects the subgroup of subjects with high mediSCORE and low GRS.

The additive measures RERI, AP and SI were calculated in terms of ORs, with 95% confidence intervals (CI) and *P* values, by two methods, the delta-method and bootstrapping. When there is no interaction, i.e., the two effects are purely additive, the RERI and AP values equals 0 and SI values equals 1.

Significance level was set to 0.05. All statistical analyses were performed using Stata (16.1, StataCorp LLC, College Station, TX, USA) and R Statistical Software (v4.0.2; R Core Team 2020).

## Results

### General

**Fig. 2:**
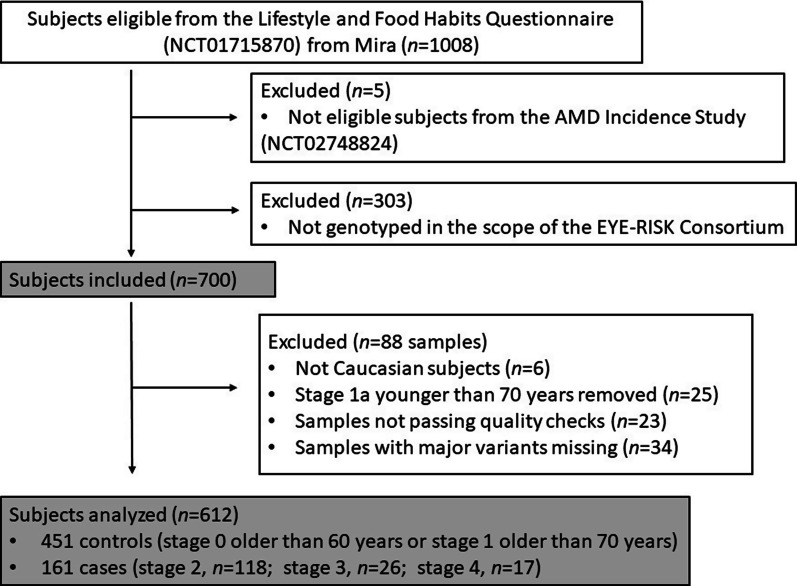
Analysis flowchart. Flowchart of the number of subjects of the Coimbra Eye Study in the analysis. n, number of subjects

Data from patients in the AMD Incidence Study, who completed a validated lifestyle and food frequency questionnaire and who were genotyped under the collaboration with the E3 were analysed. Our final sample was 612 subjects, with 161 cases and 451 controls. The analysis flowchart can be found in Fig. 2.

Demographic, behavioural, and genetic characteristics were compared between cases and controls and the results are presented in Table 1. Cases tended to be older (*P* < 0.001), were less likely to do physical exercise (*P* = 0.008), presented with a lower adherence to the Mediterranean diet, indicated by a lower mediSCORE (*P* < 0.001) and a higher GRS (*P* < 0.001). When assessing each group belonging to the mediSCORE (Table 1), intake of vegetables, fruits and dairy products was significantly different between the two groups. Vegetables and fruit consumption was superior in controls (*P* < 0.001), whereas dairies intake was superior in cases (*P* = 0.003).

**Table 1:** General characteristics (demographic, behavioural and genetic) between cases and controls

Parameters	Cases (n = 161)	Controls (n = 451)	*P* value
Gender, n (%)			0.120
Female	101 (62.7%)	251 (55.7%)	
Male	60 (37.3%)	200 (44.3%)	
Age, mean (SD)	74.8 (6.8)	71.8 (6.4)	**< 0.001**
Smoking, n (%)			0.600
Non-smoker	136 (84.5%)	386 (86.0%)	
Smoker/ex-smoker	25 (15.5%)	63 (14.0%)	
Obesity, n (%)			0.140
BMI < 25 kg/m^2^	47 (29.4%)	106 (23.5%)	
BMI ≥ 25 kg/m^2^	113 (70.6%)	345 (76.5%)	
Educational status, n (%)			0.200
Basic education	136 (84.5%)	382 (84.7%)	
High school	10 (6.2%)	36 (8.0%)	
Higher education	3 (1.9%)	16 (3.5%)	
Without education	12 (7.5%)	17 (3.8%)	
Physical exercise, n (%)			**0.008**
No	104 (64.6%)	237 (52.5%)	
Yes	57 (35.4%)	214 (47.5%)	
Genetic risk score, n (%)			**< 0.001**
Low GRS	62 (38.5%)	244 (54.1%)	
High GRS	99 (61.5%)	207 (45.9%)	
mediSCORE, n (%)			**< 0.001**
High	15 (9.3%)	105 (23.3%)	
Low	146 (90.7%)	346 (76.7%)	
mediSCORE components^a^, n (%)			
Vegetables	62 (38.5%)	243 (53.9%)	**< 0.001**
Legumes	77 (47.8%)	249 (55.2%)	0.110
Fruits	59 (36.6%)	250 (55.4%)	**< 0.001**
Cereals	90 (55.9%)	225 (49.9%)	0.200
Fish	90 (55.9%)	221 (49.0%)	0.130
Dairy products	98 (60.9%)	214 (47.5%)	**0.003**
Meat	87 (54.0%)	233 (51.7%)	0.600
Alcohol	14 (8.7%)	36 (8.0%)	0.800
Ratio of monounsaturated lipids/saturated lipids	71 (44.1%)	214 (47.5%)	0.500

Bold values represent statistically significant differences between cases and controls with *P* < 0.05, using the Pearson's Chi-squared test (or Fisher's exact test, when appropriate) for categorical variables and the Mann–Whitney U Test for continuous variables

*n* = number of subjects; *SD* = standard deviation; *BMI* = body mass index; *GRS* = genetic risk score; *mediSCORE* = adherence to the Mediterranean diet score

^a^Percentage of subjects with food group consumption above (for beneficial food groups–vegetables, legumes, fruits, cereals, fish, ratio of monounsaturated lipids to saturated lipids and moderate alcohol intake) and below (for detrimental food groups–meat and dairy products) the sex-specific median for Mira population

The minor allele frequencies (MAF) of the major common risk variants that enable the calculation of the GRS are also described as a percentage in Additional file 3: Table S3. The SNPs rs3750846, (*ARMS2/HTRA1)* and *rs570618* (*CFH)* are more frequent in cases, while the other SNPs, rs10922109 (*CFH*), rs429608 (*C2/CFB/SKIV2L*) and rs2230199 (*C3*) are more frequent in controls. These major risk variants are in the complement system and *ARMS2* genes, that are highly associated with AMD [19].

### GRS

**Fig. 3:**
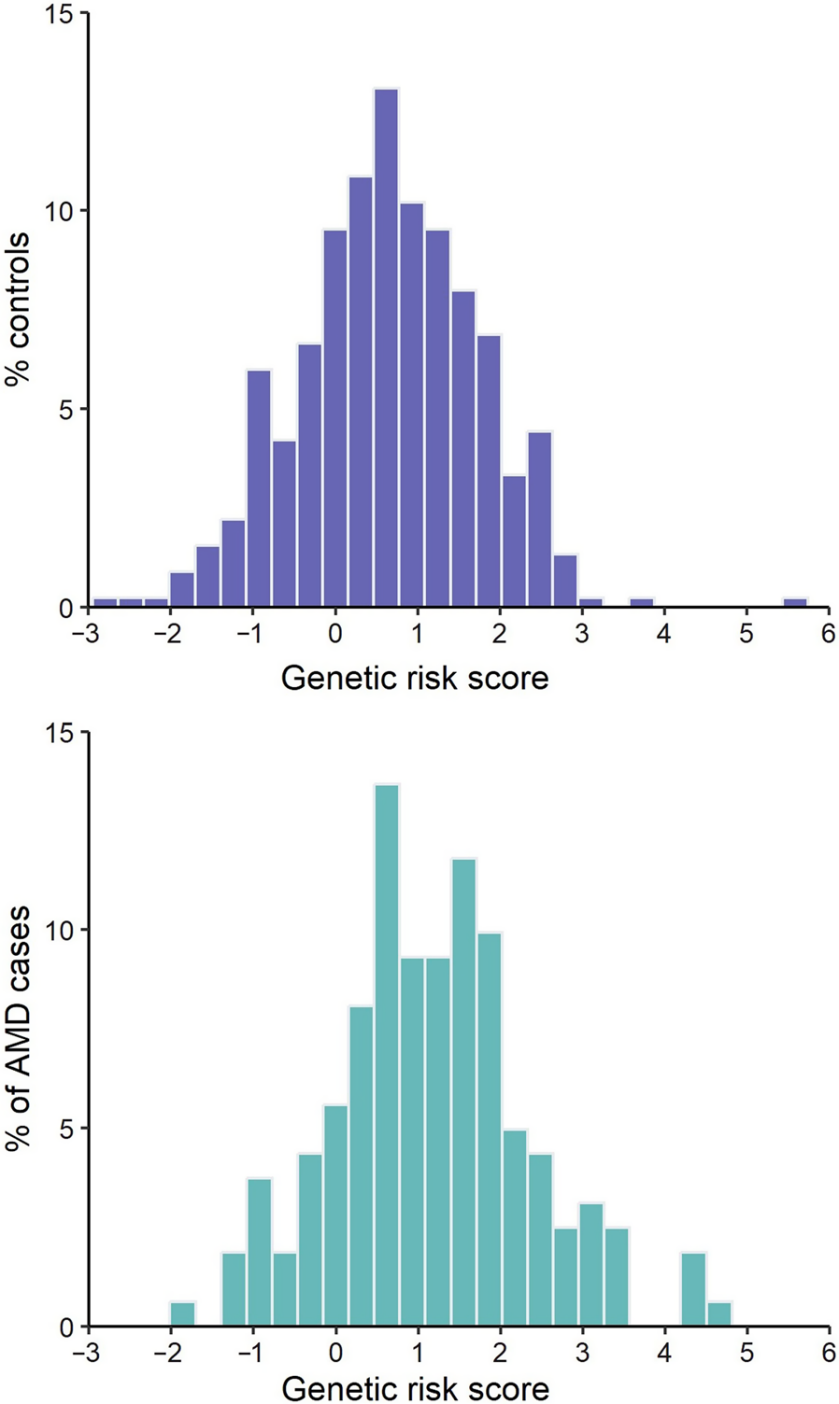
Genetic risk score distribution between controls (top) and cases (bottom). AMD, age-related macular degeneration

The distribution of the GRS of the population, by cases and controls, is presented in Fig. 3. The GRS is significantly different between cases and controls (1.173 ± 1.984 for cases and 0.640 ± 1.083 for controls, *P* < 0.001). It varies from − 2.905 to 5.526 in cases and from − 1.717 to 4.737 in controls.

### Associations between mediSCORE and AMD

The associations of AMD with the mediSCORE and the GRS are presented in Table 2.

**Table 2:** Association of AMD with the GRS and the mediSCORE (N = 1216 eyes)

Parameters	OR^a^	95% CI	*P* value
Genetic risk score			
Low GRS	1.000	–	Ref
High GRS	1.963	1.344, 2.866	**< 0.001**
mediSCORE			
Low mediSCORE	1.000	–	Ref
High mediSCORE	0.406	0.226, 0.728	**0.002**
Gender			
Female	1.000	**–**	Ref
Male	0.514	0.329, 0.803	**0.003**
Age, years			
60–70 years	1.000	–	Ref
70–75 years	1.467	0.887, 2.425	0.136
> 75 years	2.839	1.806, 4.463	**< 0.001**
Physical exercise			
No	1.000	–	
Yes	0.688	0.465, 1.017	0.061
Smoking			
Non-smoker	1.000	–	
Smoker/ex-smoker	2.072	1.149, 3.736	**0.015**

*AMD* = age-related macular degeneration; *OR* = odds ratio; *CI* = confidence interval; *GRS* = genetic risk score; *mediSCORE* = adherence to the Mediterranean diet score; *ref* = reference

Bold values represent statistically significant OR with *P* < 0.05

^a^ORs (95% CIs) were estimated by using logistic regression model with generalized estimating equations (GEE) with individual eye as the unit of analysis, adjusted for age, sex, smoking and physical exercise. The reference categories in each variable are indicated in the table above as ref

The model was adjusted for age, sex, physical exercise, and smoking. A high adherence to the Mediterranean diet showed to be protective for AMD, reducing the risk 60% (OR = 0.406, 95% CI: 0.226 to 0.728, *P* = 0.002).

High GRS (OR = 1.963, 95% CI: 1.344 to 2.866, *P* < 0.001), advanced age (OR = 2.839, 95% CI: 1.806 to 4.463, *P* < 0.001) and present or past smoking habits (OR = 2.072, 95% CI: 1.149 to 3.736, *P* = 0.015) were statistically significant in association with AMD. Being male (OR = 0.514, 95% CI: 0.329 to 0.803, *P* = 0.003) presented as protective against AMD. Performing physical exercise did not associate significantly to AMD, even though a slight protection for disease was shown (OR = 0.688, 95% CI: 0.465 to 1.017, *P* = 0.061).

### Interplay of the GRS and adherence to the Mediterranean diet

We assessed the interaction between the adherence to the Mediterranean diet and GRS in the risk for disease. Interaction was calculated using both multiplicative and additive scales (Tables 3, 4).

**Table 3:** Combined effects between mediSCORE and GRS on AMD risk (N = 1216 eyes)

	Low GRS (0)		High GRS (1)		OR^a^ (95% CI) for GRS within strata of mediSCORE
	N (cases/controls)	OR^a^ (95% CI)	N (cases/controls)	OR^a^ (95% CI)	
High mediSCORE (0)	10/125	1.000 (reference)	13/91	1.788 (0.594–5.381) *P* = 0.301	2.037 (0.658–6.301) *P* = 0.217
Low mediSCORE (1)	83/390	2.327 (0.948–5.714) *P* = 0.065	145/359	4.624 (1.908–11.202) ***P*** **< 0.001**	1.980 (1.329–2.949) ***P*** **< 0.001**
ORs^a^ (95% CI) for mediSCORE within strata of GRS		2.296 (0.933–5.651) *P* = 0.070		2.589 (1.218–5.503) ***P*** **= 0.013**	

*AMD* = age-related macular degeneration; *OR* = odds ratio; *CI* = confidence interval; *GRS* = genetic risk score; *mediSCORE* = adherence to the Mediterranean diet score; *N* = number of subjects

Bold values represent statistically significant OR with *P* < 0.05

^a^ORs (95% CIs) were estimated by using logistic regression model with generalized estimating equations (GEE) with individual eye as the unit of analysis, adjusted for age, sex, smoking and physical exercise. The reference category (high mediSCORE and low GRS) was chosen to assure that all factors are risk factors since additive measures were developed to use only with risk factors

**Table 4:** Risk for AMD due to multiplicative and additive interaction of GRS and mediSCORE

Scale of interaction	Metric	Value (95% CI)
Multiplicative	OR	1.111 (0.346–3.569); *P* = 0.859
Additive	RERI = OR_11_ − OR_01_ − OR_10_ + 1	1.509 (− 0.414–3.432); *P* = 0.062
	AP = RERI/OR_11_	0.326 (− 0.074–0.726); *P* = 0.055
	SI=(OR_11_ − 1)/[(OR_01_ − 1) + (OR_10_ − 1)]	1.713 (0.098–3.329); ***P*** **= 0.019**

*AMD* = age-related macular degeneration; *OR* = odds ratio; *CI* = confidence interval; *GRS* = genetic risk score; *mediSCORE* = adherence to the Mediterranean diet score; *RERI* = relative excess risk due to interaction; *AP* = attributable proportion; *SI* = synergy index

Bold values represent statistically significant OR with *P* < 0.05

OR_01_ refers to the risk for AMD of subjects with high adherence to the Mediterranean diet and high GRS

OR_10_ refers to the risk for AMD of subjects with low adherence to the Mediterranean diet and low GRS

OR_11_ refers to the risk for AMD of subjects with low adherence to the Mediterranean diet and high GRS

**Fig. 4:**
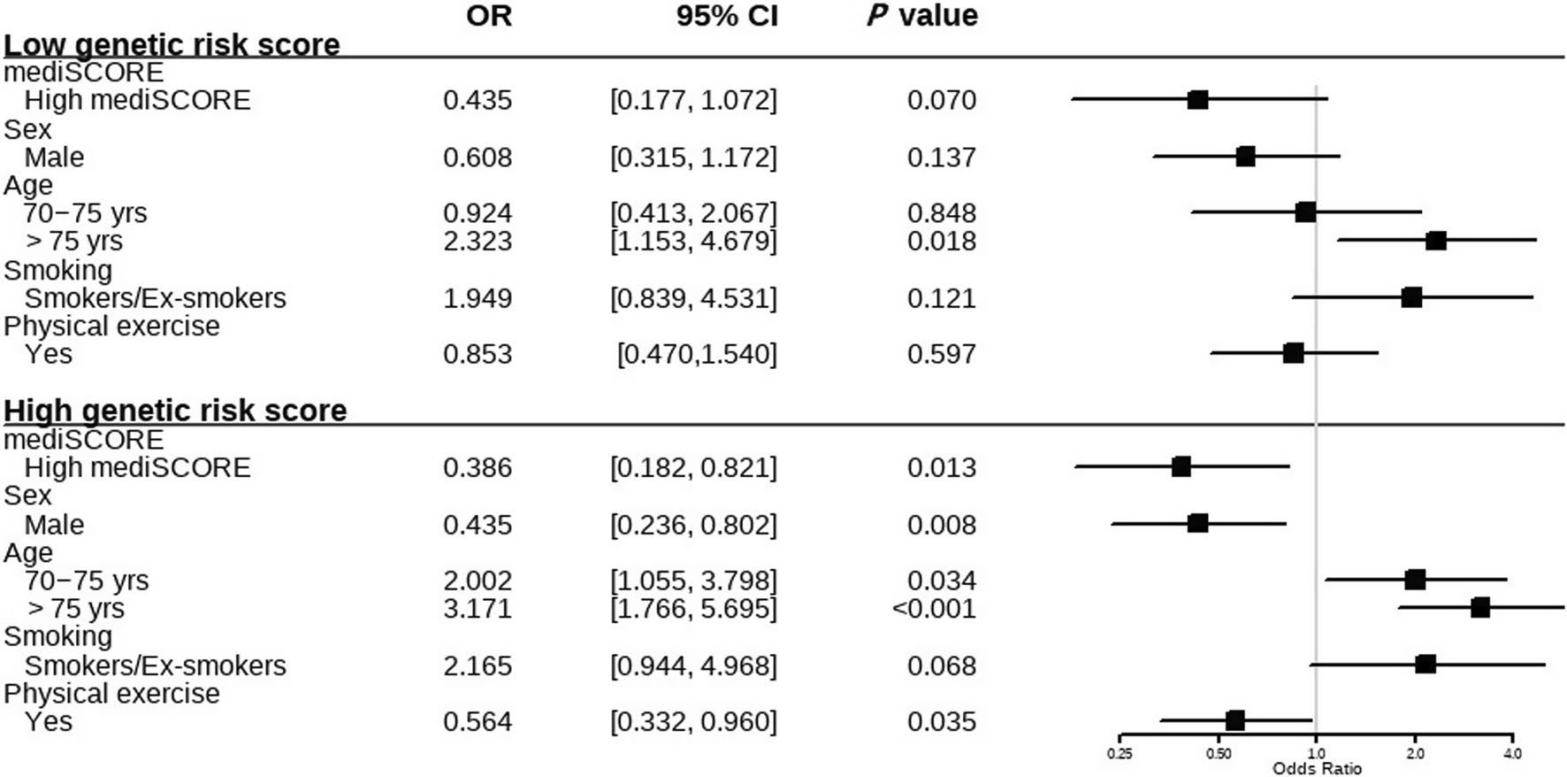
Effect of the adherence to the Mediterranean diet on AMD stratified by genetic risk score. ORs (95% CIs) were estimated by using logistic regression model with generalized estimating equations (GEE) with individual eye as the unit of analysis, adjusted for age, sex, smoking and physical exercise. OR, odds ratio; CI, confidence interval; mediSCORE, adherence to the Mediterranean diet score; yrs, years

Both factors individually indicate risk for AMD (OR = 2.327, 95% CI: 0.948–5.714, *P* = 0.065 for low mediSCORE; OR = 1.788, 95% CI: 0.594–5.381, *P* = 0.301 for high GRS), after recoding high mediSCORE as protective effect. The combined effects of having a low adherence to the Mediterranean diet and a high GRS presented nearly a 5-fold increase in the risk for AMD, when compared to a low GRS and a high adherence to the Mediterranean diet (OR = 4.624, 95% IC: 1.908–11.202, *P* = 0.0007). A measure of interaction on the multiplicative scale (OR = 1.111, 95% CI: 0.346–3.569, *P* = 0.859) suggests a multiplicative interaction, although not a statistically significant one (Table 4). The table also shows the association between mediSCORE and AMD within strata of the GRS and the same information is depicted as a forest plot in Fig. 4. For sake of clarity, the ORs are coded as usual in Fig. 4. High GRS people benefited from adhering to the Mediterranean diet with a 60% risk reduction (OR = 0.386, 95%CI: 0.182–0.821, *P* = 0.013). For low-GRS subjects, a risk reduction was also seen, but was not statistically significant (OR = 0.435, 95% CI: 0.177–1.072, *P* = 0.070). In the group of subjects with high GRS, age increased the risk for AMD by 2-fold for ages 70–75 years (*P* = 0.034) and by 3-fold for ages over 75 years (*P* < 0.001). The same was seen for smoking (OR = 2.165), though not significantly (*P* = 0.068). Performing any type of physical exercise also presented as a protective factor (OR = 0.564, *P* = 0.035).

The additive model showed a causal effect of the interaction of GRS and adherence to the Mediterranean diet. The measures of interaction on the additive model give consistent results as each measure (RERI, AP and SI) indicates positive interaction on an additive scale, proving that the effect on the risk for disease of this interaction was superior to the effect of each factor in separate, with a RERI of 150.9% (95% CI: − 0.414 to 3.432, *P* = 0.062), an AP of 0.326 (95% CI: − 0.074 to 0.726, *P* = 0.055) and an SI of 1.713 (95% CI: 0.098–3.329, *P* = 0.019).

## Discussion

A lower adherence to the Mediterranean diet was associated with an AMD risk 2.5 times higher (Table 2), in the overall population, controlling for age, sex, GRS, physical exercise, and smoking. The combined effects of having a low adherence to the Mediterranean diet and a high GRS led to a higher than 4-fold increased risk of AMD, compared to a high adherence to the Mediterranean diet and a low GRS.

Mediterranean diet has been widely associated with a decreased risk for AMD and progression, mainly due to a higher intake of fruits, vegetables, olive oil, whole-grain, and fish [33, 34]. This dietary pattern is rich in antioxidants, unsaturated fats, lutein, and zeaxanthin [35, 36], which have been regarded as protective for AMD due to their antithrombotic and anti-inflammatory roles [37, 38]. The healthy retina naturally consumes energy at a high rate due to its high metabolism, compared with other organs. Energy consumption is required to perform phototransduction and to maintain the integrity and active function of photoreceptors, and this high energy demand state leads to oxidative stress, even when no underlying diseases are present [39]. The redox system in the retina is of utmost importance for retinal metabolism. An enhanced oxidative stress environment leads to mitochondrial dysfunction and, hence, to the loss of photoreceptors, accumulation of cellular debris and macular atrophy, which are disease drivers. Antioxidants, fatty acids, and carotenoids (lutein and zeaxanthin) contribute to the preservation of the retinal membrane of the photoreceptors and the retinal pigment epithelium cells, along with the maintenance of the antioxidant system at physiological levels [40–43].

Assessing a dietary pattern rather than the intake of single nutrients is advantageous, since nutrients may present biological interactions and synergistic effects that may extrapolate their isolated effect [44]. This is particularly important in a complex disease such as AMD, with different pathways involved in its pathophysiology and different risk factors contributing to the establishment of the disease. A closer adherence to the Mediterranean diet has also been associated with better disease outcomes in cardiovascular [26, 27] and neurological diseases [14]. AMD shares risk factors and pathophysiological mechanisms with cardiovascular and neurological diseases [45–47], therefore the protective effect of Mediterranean diet for AMD seems very plausible.

In our study, people with a high genetic risk significantly benefited more from adhering to the Mediterranean diet, with a decrease in the risk of 60% when compared to high genetic risk subjects who present a low adherence to the Mediterranean diet, whereas low-risk genetic subjects did not. The multiplicative model was not statistically significant, though some interaction was shown, but the additive model proved interaction between the adherence to the Mediterranean diet and the genetic risk. Our results confirm those of Merle et al. [7] that showed a statistically significant protective effect of Mediterranean diet in the progression of AMD in those people genetically at risk, in the Age-Related Eye Disease Study (AREDS) population. In both studies, ours and Merle’s, adherence to the Mediterranean diet did seem beneficial for people with a low genetic risk, but not at a statistically significant level. Other reports have shown results with some similarity. A large study from the EYE-RISK Consortium [33] reported that, despite the gain of a healthy lifestyle by the population in general, those people with a high GRS for AMD gained more, with a higher risk reduction for AMD: from OR = 35.03 (unfavourable lifestyle) to OR = 14.90 (favourable lifestyle).

Correspondingly, the risk for late AMD increased with an unhealthy lifestyle but this risk was found to be more pronounced in people with a higher GRS. A healthy lifestyle was defined as absence of smoking habits and high intake of vegetables, fruits and fish, which resembles part of a Mediterranean diet. Also, vegetables, fruits and fish are rich in antioxidants and unsaturated fats. Indeed, the intake of antioxidants and omega3 fatty acids was reported to reduce the risk of early AMD and GA in people at high genetic risk for AMD conferred by the risk alleles of *CFH* rs1061170 and *ARMS2* rs10490924, confirming that the benefit of a healthy diet increases in individuals with a higher genetic risk for AMD [48, 49]. In our study, performing physical exercise was protective against AMD in people at genetic risk, which supports the idea of a healthy lifestyle being associated with a decreased risk particularly in these subjects. We must, however, acknowledge that AMD patients who suffer vision loss may present more difficulty in performing physical exercise than controls do.

We used GRS to define the genetic risk for each subject. This GRS is based on the 52 variants identified by IAMDGC [19], both rare and common, which are considered the hallmarks of AMD genetics. In case of absence of any of the five major variants, the GRS was considered null. Regarding these variants, in our population only two statistically significant associations were found: *CFH* rs10922109 and *C2/CFB/SKIV2L* rs429608. They were more predominant in controls than in cases, suggesting that they might be protective for AMD, as shown by Fritsche and the EYE-RISK Consortium: *CFH* rs10922109 OR = 0.37, *P* = 3.93^− 47^ (EYE-RISK Consortium) and OR = 0.38 *P* = 9.6^− 618^ (IAMDGC) and *C2/CFB/SKIV2L* rs429608 OR = 0.62, *P* = 1.00^− 6^ (EYE-RISK Consortium) and OR = 0.57 *P* = 1.2^− 103^ (IAMDGC) [19, 30]. A higher GRS signifies a higher probability of having AMD, depicting a shift to the right (Fig. 3). A high GRS increased the risk of AMD almost 2-fold, and the GRS was significantly different from cases and controls. However, we must acknowledge that the two GRS distribution figures show an overlap, implying there is an impossibility to distinguish between the two groups based on GRS alone. Other study groups have reported similar results on this overlap, suggesting the genetic risk for AMD is widespread in the overall population [30, 33, 34]. As it was demonstrated by The EYE-RISK Consortium [33], the complement pathway and *ARMS2* alone confer at least 90% of the genetic burden for late AMD, but genes from other pathways also account for genetic burden, highlighting the polygenic nature of AMD, and explaining the high prevalence of genetic susceptibility for AMD. Likewise, the fact that the genetic background by itself does not discern clearly between having the disease or not emphasizes the multifactorial essence of AMD. In our risk model, present or past smoking habits were a risk factor for AMD, increasing the risk for disease more than 2-fold. Physical exercise, however, did not show a statistically significant protection for the disease, although it was able to reduce the risk, unlike other studies [50, 51]. The typical AMD patient, in our study, is older, performs less exercise, has a higher GRS and a lower adherence to Mediterranean diet, consuming fewer vegetables and fruits, and more dairy products.

Even though our results and Merle’s study [7] results are similar, we must emphasise that our GRS is composed of 52 SNPs, against 10 of Merle’s. This raises the attention to the fact that a thorough GRS calculation, with all the genes previously associated with the disease, is indeed needed in a context of interaction of Mediterranean diet and genetics. An AREDS investigation [8] suggested that the *CFH* gene might be more important in influencing the protective effect of the Mediterranean diet and the risk for AMD compared to the whole genetic burden that balances the causal and protective roles of genes.

Given the differences found in the two GRS strata, we then assessed the association between the adherence to the Mediterranean diet and the genetic risk for disease by multiplicative and additive interaction. No statistically significant interaction was seen for multiplicative interaction, but the additive interaction model presented interesting results. The risk for AMD obtained by the addition of two factors separately was inferior to the risk for AMD obtained by addition of the interplay between them, suggesting that genetics reshapes the outcome of the diet and, therefore, of its properties. Whether genetics influences the effects of diet, or the diet influences genetic expression patterns, having, therefore, an influence on genetic risk, or both factors influence one another, is still unknown. The fact that the SI proved statistical significance is worth mention, given its importance in additive versus multiplicative models in clinical research. Additive models translate synergies of risk factors in a biological context, rather than multiplicative interactions, since factors may add to one another instead of multiplying each individual action. This idea has also been studied in cardiovascular diseases, concluding that the response to dietary salt on hypertension depends on the genetic susceptibility [52, 53].

We must acknowledge that our study has limitations, like the relatively small sample and the fact that we are studying a cohort of a specific region of Portugal, which may shape the genetic analysis. Also, as the Coimbra Eye Study is a population-based study, our sample is not sex- and age-matched. Additionally, as this is a cross-sectional study, we cannot assume causality in adherence to the Mediterranean diet and disease. As strengths, we highlight the well phenotyped population with a multimodal grading by expert medical graders and the validated questionnaires and score for the assessment of the Mediterranean diet. In fact, the mediSCORE has been validated for cardiovascular diseases, as previously mentioned, and results on both AMD and cardiovascular seem to relate to a common path.

## Conclusions

Weighting the individual genetic risk for disease may be beneficial to assess the benefit of non-pharmacological personalized strategies. This is particularly important when non-pharmacological strategies rely on modifiable risk factors to delay AMD or its progression.

The significant benefit of a high adherence to the Mediterranean diet in high genetic risk subjects seems to lie on an additive model translating synergies of these factors. Further studies should be conducted in the future with larger populations to clarify the gene-environment effect.

### Supplementary Information

**Additional file 1**: **Table S1.** Scoring system for the model of adherence to the Mediterranean diet (mediSCORE)–sex-specific medians are presented for the coastal town (Mira n=1008).

**Additional file 2**: **Table S2**. AMD variants used in the GRS calculation in the CES study.

**Additional file 3**: **Table S3**. Genetic characteristics of the major risk variants associated with AMD.

## Data Availability

The datasets used and/or analysed during the current study are available from the corresponding author on reasonable request.

## Abbreviations

*ABCA4*: ATP binding cassette subfamily A member 4
AIBILI: Association for Innovation and Biomedical Research on Light and Image
AMD: Age-related macular degeneration
AP: Attributable proportion due to interaction
AREDS: Age-Related Eye Disease Study
*ARMS2*: Age-related maculopathy susceptibility 2
*CD46*: Cluster of differentiation 46
CES: Coimbra Eye Study
CI: Confidence intervals
*CFB*: Complement factor B
*CFH*: Complement factor H
*CFI*: Complement factor I
CFP: Color fundus photography
CNV: Choroidal neovascularization
CORC: Coimbra Ophthalmology Reading Center
*CTNNA1*: Catenin Alpha 1
*C3*: Complement component 3
*C9*: Complement component 9
ETDRS: Early Treatment Diabetic Retinopathy Study
E3: European Eye Epidemiology Consortium
FAF: Fundus autofluorescence
GA: Geographic atrophy
GRS: Genetic risk score
GWAS: Genome-wide association studies
*HTRA1*: HtrA Serine Peptidase 1
IAMDGC: International Age-related Macular Degeneration Genomics Consortium
IR: Infra-red
KASP: Kompetitive allele specific polymerase chain reaction
OR: Odds ratio
*PRPH2*: Peripherin 2
RERI: Relative excess risk due to interaction
SD-OCT: Spectral domain optical coherence tomography
SI: Synergy index
*SKIV2L*: Ski2-like RNA Helicase
*SLC16A8*: Solute carrier family 16 member 8
SNPs: Single nucleotide polymorphisms
STOBE: Strengthening the Reporting of Observational Study
*TIMP3*: Tissue inhibitor of metalloproteinase-3

## References

[1] Colijn JM, Buitendijk GHS, Prokofyeva E, Alves D, Cachulo ML, Khawaja AP (2017). Prevalence of age-related macular degeneration in Europe: the past and the future. Ophthalmology.

[2] Wong WL, Su X, Li X, Cheung CM, Klein R, Cheng CY (2014). Global prevalence of age-related macular degeneration and disease burden projection for 2020 and 2040: a systematic review and meta-analysis. Lancet Glob Health.

[3] Lambert NG, ElShelmani H, Singh MK, Mansergh FC, Wride MA, Padilla M (2016). Risk factors and biomarkers of age-related macular degeneration. Prog Retin Eye Res.

[4] Chakravarthy U, Wong TY, Fletcher A, Piault E, Evans C, Zlateva G (2010). Clinical risk factors for age-related macular degeneration: a systematic review and meta-analysis. BMC Ophthalmol.

[5] Raimundo M, Mira F, Cachulo MDL, Barreto P, Ribeiro L, Farinha C (2018). Adherence to a Mediterranean diet, lifestyle and age-related macular degeneration: the Coimbra Eye study–report 3. Acta Ophthalmol.

[6] Nunes S, Alves D, Barreto P, Raimundo M, da Luz Cachulo M, Farinha C (2018). Adherence to a Mediterranean diet and its association with age-related macular degeneration. The Coimbra Eye study—report 4. Nutrition.

[7] Merle BM, Silver RE, Rosner B, Seddon JM (2015). Adherence to a Mediterranean diet, genetic susceptibility, and progression to advanced macular degeneration: a prospective cohort study. Am J Clin Nutr.

[8] Keenan TD, Agrón E, Mares J, Clemons TE, van Asten F, Swaroop A (2020). Adherence to the Mediterranean diet and progression to late age-related macular degeneration in the Age-Related Eye Disease Studies 1 and 2. Ophthalmology.

[9] Merle BMJ, Colijn JM, Cougnard-Grégoire A, de Koning-Backus APM, Delyfer MN, Kiefte-de Jong JC (2019). Mediterranean diet and incidence of advanced age-related macular degeneration: the EYE-RISK Consortium. Ophthalmology.

[10] Hogg RE, Woodside JV, McGrath A, Young IS, Vioque JL, Chakravarthy U (2017). Mediterranean diet score and its association with age-related macular degeneration: the European Eye Study. Ophthalmology.

[11] Piermarocchi S, Tognetto D, Piermarocchi R, Masetto M, Monterosso G, Segato T (2016). Risk factors and age-related macular degeneration in a Mediterranean-basin population: the PAMDI (prevalence of age-related Macular Degeneration in Italy) Study-Report 2. Ophthalmic Res.

[12] Guasch-Ferré M, Willett WC (2021). The Mediterranean diet and health: a comprehensive overview. J Intern Med.

[13] Tang C, Wang X, Qin LQ, Dong JY (2021). Mediterranean diet and mortality in people with cardiovascular disease: a meta-analysis of prospective cohort studies. Nutrients.

[14] Keenan TD, Agrón E, Mares JA, Clemons TE, van Asten F, Swaroop A (2020). Adherence to a Mediterranean diet and cognitive function in the Age-Related Eye Disease Studies 1 & 2. Alzheimers Dement.

[15] Mentella MC, Scaldaferri F, Ricci C, Gasbarrini A, Miggiano GAD (2019). Cancer and Mediterranean diet: a review. Nutrients.

[16] Morze J, Danielewicz A, Przybyłowicz K, Zeng H, Hoffmann G, Schwingshackl L (2021). An updated systematic review and meta-analysis on adherence to mediterranean diet and risk of cancer. Eur J Nutr.

[17] Willett WC, Sacks F, Trichopoulou A, Drescher G, Ferro-Luzzi A, Helsing E (1995). Mediterranean diet pyramid: a cultural model for healthy eating. Am J Clin Nutr.

[18] Serra R, Rallo V, Pinna A, Steri M, Piras MG, Marongiu M (2023). Polygenic risk score and biochemical/environmental variables predict a low-risk profile of age-related macular degeneration in Sardinia. Graefes Arch Clin Exp Ophthalmol.

[19] Fritsche LG, Igl W, Bailey JN, Grassmann F, Sengupta S, Bragg-Gresham JL (2016). A large genome-wide association study of age-related macular degeneration highlights contributions of rare and common variants. Nat Genet.

[20] Fritsche LG, Chen W, Schu M, Yaspan BL, Yu Y, Thorleifsson G (2013). Seven new loci associated with age-related macular degeneration. Nat Genet.

[21] Geerlings MJ, de Jong EK, den Hollander AI (2017). The complement system in age-related macular degeneration: a review of rare genetic variants and implications for personalized treatment. Mol Immunol.

[22] Cachulo Mda L, Lobo C, Figueira J, Ribeiro L, Laíns I, Vieira A (2015). Prevalence of age-related macular degeneration in Portugal: the Coimbra Eye Study—Report 1. Ophthalmologica.

[23] Cachulo Mda L, Laíns I, Lobo C, Figueira J, Ribeiro L, Marques JP (2016). Age-related macular degeneration in Portugal: prevalence and risk factors in a coastal and an inland town. The Coimbra Eye study – report 2. Acta Ophthalmol.

[24] Farinha CVL, Cachulo ML, Alves D, Pires I, Marques JP, Barreto P (2019). Incidence of age-related macular degeneration in the central region of Portugal: the Coimbra Eye Study-Report 5. Ophthalmic Res.

[25] Lopes C. Reprodutibilidade e validação de um questionário semi-quantitativo de frequência alimentar. In: Alimentação e enfarte agudo do miocárdio: estudo caso controlo de base comunitária. [Porto]: University of Porto; 2000. Available at https://repositorio-aberto.up.pt/bitstream/10216/9938/2/2734_TD_01_C.pdf. Accessed 24 Jul 2023.

[26] Trichopoulou A, Costacou T, Bamia C, Trichopoulos D (2003). Adherence to a Mediterranean diet and survival in a greek population. N Engl J Med.

[27] Trichopoulou A, Kouris-Blazos A, Wahlqvist ML, Gnardellis C, Lagiou P, Polychronopoulos E (1995). Diet and overall survival in elderly people. BMJ.

[28] Vingerling JR, Dielemans I, Hofman A, Grobbee DE, Hijmering M, Kramer CF (1995). The prevalence of age-related maculopathy in the Rotterdam Study. Ophthalmology.

[29] Klaver CC, Assink JJ, van Leeuwen R, Wolfs RC, Vingerling JR, Stijnen T (2001). Incidence and progression rates of age-related maculopathy: the Rotterdam Study. Invest Ophthalmol Vis Sci.

[30] de Breuk A, Acar IE, Kersten E, Schijvenaars MMVAP, Colijn JM, Haer-Wigman L (2021). Development of a genotype assay for age-related macular degeneration: the EYE-RISK Consortium. Ophthalmology.

[31] Knol MJ, VanderWeele TJ, Groenwold RHH, Klungel OH, Rovers MM, Grobbee DE (2011). Estimating measures of interaction on an additive scale for preventive exposures. Eur J Epidemiol.

[32] Knol MJ, VanderWeele TJ (2012). Recommendations for presenting analyses of effect modification and interaction. Int J Epidemiol.

[33] Colijn JM, Meester-Smoor M, Verzijden T, de Breuk A, Silva R, Merle BMJ (2021). Genetic risk, lifestyle, and age-related macular degeneration in Europe: the EYE-RISK Consortium. Ophthalmology.

[34] Merle BMJ, Rosner B, Seddon JM (2020). Genetic susceptibility, diet quality, and two-step progression in drusen size. Invest Ophthalmol Vis Sci.

[35] Dai J, Jones DP, Goldberg J, Ziegler TR, Bostick RM, Wilson PW (2008). Association between adherence to the Mediterranean diet and oxidative stress. Am J Clin Nutr.

[36] Cougnard-Grégoire A, Merle BM, Rougier JF, Rougier MB, Delyfer MN, Le Goff M (2016). Olive oil consumption and age-related macular degeneration: the Alienor Study. PLoS ONE.

[37] de Koning-Backus APM, Buitendijk GHS, Kiefte-de Jong JC, Colijn JM, Hofman A, Vingerling JR (2019). Intake of vegetables, fruit, and fish is beneficial for age-related macular degeneration. Am J Ophthalmol.

[38] Nani A, Murtaza B, Khan AS, Khan NA, Hichami A (2021). Antioxidant and anti-inflammatory potential of polyphenols contained in Mediterranean diet in obesity: molecular mechanisms. Molecules.

[39] Ozawa Y (2020). Oxidative stress in the light-exposed retina and its implication in age-related macular degeneration. Redox Biol.

[40] Lem DW, Davey PG, Gierhart DL, Rosen RB (2021). A systematic review of carotenoids in the management of age-related macular degeneration. Antioxid (Basel).

[41] van Leeuwen EM, Emri E, Merle BMJ, Colijn JM, Kersten E, Cougnard-Gregoire A (2018). A new perspective on lipid research in age-related macular degeneration. Prog Retin Eye Res.

[42] Chua B, Flood V, Rochtchina E, Wang JJ, Smith W, Mitchell P (2006). Dietary fatty acids and the 5-year incidence of age-related maculopathy. Arch Ophthalmol.

[43] Chew EY, Clemons TE, Agrón E, Domalpally A, Keenan TDL, Vitale S (2022). Long-term outcomes of adding lutein/zeaxanthin and ω-3 fatty acids to the AREDS supplements on age-related macular degeneration progression: AREDS2 report 28. JAMA Ophthalmol.

[44] Ocké MC (2013). Evaluation of methodologies for assessing the overall diet: dietary quality scores and dietary pattern analysis. Proc Nutr Soc.

[45] Tan JS, Wang JJ, Liew G, Rochtchina E, Mitchell P (2008). Age-related macular degeneration and mortality from cardiovascular disease or stroke. Br J Ophthalmol.

[46] Snow KK, Seddon JM (1999). Do age-related macular degeneration and cardiovascular disease share common antecedents?. Ophthalmic Epidemiol.

[47] Rong SS, Lee BY, Kuk AK, Yu XT, Li SS, Li J (2019). Comorbidity of dementia and age-related macular degeneration calls for clinical awareness: a meta-analysis. Br J Ophthalmol.

[48] Ho L, van Leeuwen R, Witteman JCM, van Duijn CM, Uitterlinden AG, Hofman A (2011). Reducing the genetic risk of age-related macular degeneration with dietary antioxidants, zinc, and ω-3 fatty acids: the Rotterdam Study. Arch Ophthalmol.

[49] Reynolds R, Rosner B, Seddon JM (2013). Dietary omega-3 fatty acids, other fat intake, genetic susceptibility, and progression to incident geographic atrophy. Ophthalmology.

[50] McGuinness MB, Le J, Mitchell P, Gopinath B, Cerin E, Saksens NTM (2017). Physical activity and age-related macular degeneration: a systematic literature review and meta-analysis. Am J Ophthalmol.

[51] Mauschitz MM, Schmitz MT, Verzijden T, Schmid M, Thee EF, Colijn JM (2022). Physical activity, incidence, and progression of age-related macular degeneration: a multicohort study. Am J Ophthalmol.

[52] Kwon YJ, Kim JO, Park JM, Choi JE, Park DH, Song Y (2020). Identification of genetic factors underlying the association between sodium intake habits and hypertension risk. Nutrients.

[53] Chu C, Wang Y, Ren KY, Yan DY, Guo TS, Zheng WL (2016). Genetic variants in adiponectin and blood pressure responses to dietary sodium or potassium interventions: a family-based association study. J Hum Hypertens.

