# Supplementary material for: Interaction between genetics and the adherence to the Mediterranean diet: the risk for age-related macular degeneration. Coimbra Eye Study Report 8
Source: Eye Vis (Lond). 2023 Aug 14;10:38. doi: 10.1186/s40662-023-00355-0 (PMC10424352; doi:10.1186/s40662-023-00355-0)
Supplement: Supplementary file 1 — Additional file 1: Table S1. Scoring system for the model of adherence to the Mediterranean diet (mediSCORE)–sex-specific medians are presented for the coastal town (Mira n=1008). [file 40662_2023_355_MOESM1_ESM.docx]

**Supplementary Table 1.** Scoring system for the model of adherence to the Mediterranean diet (mediSCORE)–sex-specific medians are presented for the coastal town (Mira n=1008).

| mediSCORE | Cut-off for 0 | | Cut-off for 1 | |
| --- | --- | --- | --- | --- |
|  | Women | Men | Women | Men |
| Vegetables^*^, g/day | < 204.4 | < 211.2 | ≥ 204.4 | ≥ 211.2 |
| Legumes^†^, g/day | < 37.9 | < 38.7 | ≥ 37.9 | ≥ 38.7 |
| Fruits and nuts^‡^, g/day | < 280.6 | < 287.6 | ≥ 280.6 | ≥ 287.6 |
| Cereals^§^, g/day | < 233.3 | < 257.3 | ≥ 233.3 | ≥ 257.3 |
| Fish^װ^, g/day | < 154.0 | < 163.3 | ≥ 154.0 | ≥ 163.3 |
| Dairy products^#^, g/day | > 254.0 | > 261.1 | ≤ 254.0 | ≤ 261.1 |
| Meat^**^, g/day | > 78.6 | > 93.5 | ≤ 78.6 | ≤ 93.5 |
| Alcohol^††^, g/day | < 5 or > 25 | < 10 or > 50 | 5–25 | 10–50 |
| Ratio of monounsaturated lipids / saturated lipids | < 1.8 | < 1.7 | ≥ 1.8 | ≥ 1.7 |

**^*^**Cabbage (5 types), broccoli, cauliflower, brussels sprouts, rapini, turnip greens, spinach, green beans, green peas, lettuce, cress, onions, carrots, turnip, fresh tomatoes, green and red peppers, cucumber.

^†^Peas, beans (red, brown, fava, etc), chickpeas, lupins, lentils.

^‡^Apples, pears, oranges, tangerines, bananas, kiwis, strawberries, cherries, peaches, plums, melons, watermelons, figs, loquats, apricots, nuts.

^§^Bread (wheat, rye, barley, whole or in mixtures), oats, corn bread, direct derivatives (corn flakes), rice, potato.

^װ^Fat and lean fishes, codfish, fish preserves, squid, octopus, shellfish.

^#^Milk (whole, half or skimmed), yoghurts, cheese, ice creams, dairy-based desserts.

^**^Chicken, rabbit, turkey, cow, pork, goat, meat derivatives (ham and similar, bacon, sausages).

^††^Wine, beer, spirits.
