# Supplementary material for: Interaction between genetics and the adherence to the Mediterranean diet: the risk for age-related macular degeneration. Coimbra Eye Study Report 8
Source: Eye Vis (Lond). 2023 Aug 14;10:38. doi: 10.1186/s40662-023-00355-0 (PMC10424352; doi:10.1186/s40662-023-00355-0)
Supplement: Supplementary file 2 — Additional file 2: Table S2. AMD variants used in the GRS calculation in the CES study. [file 40662_2023_355_MOESM2_ESM.docx]

| Supplementary Table 2. AMD variants used in the GRS calculation in the CES study. | | | | | | | | | |
| --- | --- | --- | --- | --- | --- | --- | --- | --- | --- |
| Gene | Rs id | Alternative Rs id | Chr:Position | Major/minor allele | OR fully conditioned analysis* | *P* value fully conditioned* | | EYE-RISK method^‡^ | |
| *CFH* | rs10922109 |  | 1:196,704,632 | C/A | 0.51 | 1.0 x 10^–131^ | smMIP | |  |
| *CFH* | rs570618 |  | 1:196,657,064 | G/T | 1.74 | 9.2 x 10^–76^ | smMIP | |  |
| *CFH* | rs121913059 |  | 1:196,716,375 | C/T | 47.63 | 2.2 x 10^–35^ | smMIP | |  |
| *CFH* | rs148553336 |  | 1:196,613,173 | T/C | 0.31 | 8.8 x 10^–17^ | smMIP | |  |
| *CFH* | rs187328863 | rs79436252 (R^2^=1.0) | 1:196,358,288 | A/G | 1.47 | 2.8 x 10^–12^ | KASPar | |  |
| *CFH (CFHR3/CFHR1)* | rs61818925 | rs61818924 (R^2^=0.80) | 1:196,815,374 | A/T | 1.18 | 6.3 x 10^–9^ | KASPar | |  |
| *CFH* | rs35292876 |  | 1:196,706,642 | C/T | 1.54 | 9.5 x 10^–8^ | smMIP | |  |
| *CFH* | rs191281603 |  | 1:196,958,651 | C/G | 0.41 | 7.7 x 10^–7^ | smMIP | |  |
| *COL4A3* | rs11884770 |  | 2:228,086,920 | C/T | 0.92 | 2.6 x 10^–4^ | smMIP | |  |
| *ADAMTS9-AS2* | rs62247658 |  | 3:64,715,155 | T/C | 1.14 | 7.8 x 10^–11^ | smMIP | |  |
| *COL8A1* | rs140647181 |  | 3:99,180,668 | T/C | 1.85 | 1.6 x 10^–14^ | smMIP | |  |
| *COL8A1* | rs55975637 |  | 3:99,419,853 | G/A | 1.16 | 3.8 x 10^–7^ | smMIP | |  |
| *CFI* | rs10033900 |  | 4:110,659,067 | C/T | 1.15 | 1.2 x 10^–13^ | smMIP | |  |
| *CFI* | rs141853578 |  | 4:110,685,820 | C/T | 5.12 | 7.4 x 10^–12^ | smMIP | |  |
| *C9* | rs62358361 |  | 5:39,327,888 | G/T | 1.67 | 7.2 x 10^–9^ | smMIP | |  |
| *PRLR/SPEF2* | rs114092250 | rs74767144 (R^2^=0.77) | 5:35,588,257 | C/G | 0.71 | 9.5 x 10^–6^ | smMIP | |  |
| *C2/CFB/SKIV2L* | rs116503776 | rs429608 ^†^ | 6:31,930,462 | G/A | 0.51 | 5.0 x 10^–96^ | smMIP | |  |
| *C2/CFB/SKIV2L* | rs144639244 | rs2746394 ^†^ | 6:31,946,792 | G/A | 2.79 | 1.0 x 10^–32^ | smMIP | |  |
| *C2/CFB/SKIV2L (PBX2)* | rs114254831 | rs204993^†^ | 6:32,155,581 | A/G | 1.13 | 8.8 x 10^–9^ | smMIP | |  |
| *C2/CFB/SKIV2L* | rs181705462 | rs114212545 (R^2^=0.84) | 6:31,932,368 | G/A | 1.56 | 2.8 x 10^–8^ | KASPar | |  |
| *VEGFA* | rs943080 |  | 6:43,826,627 | T/C | 0.87 | 5.8 x 10^–13^ | smMIP | |  |
| *KMT2E/SRPK2* | rs1142 |  | 7:104,756,326 | C/T | 1.14 | 1.3 x 10^–10^ | KASPar | |  |
| *PILRB/PILRA* | rs7803454 |  | 7:99,991,548 | C/T | 1.15 | 2.8 x 10^–9^ | smMIP | |  |
| *TNFRSF10A* | rs79037040 |  | 8:23,082,971 | T/G | 0.89 | 5.1 x 10^–9^ | KASPar | |  |
| *MIR6130/RORB* | rs10781182 |  | 9:76,617,720 | G/T | 1.12 | 1.5 x 10^–6^ | smMIP | |  |
| *TRPM3* | rs71507014 |  | 9:73,438,605 | GC/G | 1.11 | 2.3 x 10^–8^ | KASPar | |  |
| *TGFBR1* | rs1626340 |  | 9:101,923,372 | G/A | 0.88 | 4.0 x 10^–7^ | smMIP | |  |
| *ABCA1* | rs2740488 |  | 9:107,661,742 | A/C | 0.89 | 6.0 x 10^–7^ | smMIP | |  |
| *ARHGAP21* | rs12357257 |  | 10:24,999,593 | G/A | 1.12 | 1.8 x 10^–6^ | smMIP | |  |
| *ARMS2/HTRA1* | rs3750846 |  | 10:124,215,565 | T/C | 2.93 | 6.0 x 10^–645^ | smMIP | |  |
| *RDH5/CD63* | rs3138141 |  | 12:56,115,778 | C/A | 1.18 | 4.7 x 10^–8^ | KASPar | |  |
| *ACAD10* | rs61941274 | rs61941272 (R^2^=1.0) | 12:112,116,776 | C/A | 1.60 | 3.2 x 10^–9^ | smMIP | |  |
| *B3GALTL* | rs9564692 |  | 13:31,821,240 | C/T | 0.90 | 1.0 x 10^–6^ | smMIP | |  |
| *RAD51B* | rs61985136 |  | 14:68,769,199 | T/C | 0.88 | 8.2 x 10^–10^ | KASPar | |  |
| *RAD51B* | rs2842339 |  | 14:68,986,999 | A/G | 1.18 | 3.3 x 10^–7^ | smMIP | |  |
| *LIPC* | rs2043085 |  | 15:58,680,954 | T/C | 1.15 | 7.7 x 10^–13^ | smMIP | |  |
| *LIPC* | rs2070895 |  | 15:58,723,939 | G/A | 0.86 | 1.8 x 10^–10^ | smMIP | |  |
| *CETP* | rs5817082 |  | 16:56,997,349 | C/CA | 0.87 | 2.7 x 10^–8^ | smMIP | |  |
| *CETP* | rs17231506 |  | 16:56,994,528 | C/T | 1.11 | 1.2 x 10^–6^ | smMIP | |  |
| *CTRB2/CTRB1* | rs72802342 | rs55993634  (R^2^=0.89) | 16:75,236,763 | C/G | 0.79 | 8.0 x 10^–9^ | smMIP | |  |
| *TMEM97/VTN* | rs11080055 |  | 17:26,649,724 | C/A | 0.92 | 1.5 x 10^–5^ | smMIP | |  |
| *NPLOC4/TSPAN10* | rs6565597 |  | 17:79,526,821 | C/T | 1.12 | 2.1 x 10^–7^ | smMIP | |  |
| *C3* | rs2230199 |  | 19:6,718,387 | C/G | 1.47 | 1.6 x 10^–60^ | smMIP | |  |
| *C3* | rs147859257 |  | 19:6,718,146 | T/G | 3.22 | 4.1 x 10^–26^ | smMIP | |  |
| *C3 (NRTN/FUT6)* | rs12019136 | rs17855739 (R^2^=0.95) | 19:5,831,840 | C/T | 0.74 | 4.0 x 10^–9^ | smMIP | |  |
| *CNN2* | rs67538026 | Rs113772652(R^2^=0.996) | 19:1,031,550 | C/T | 0.90 | 1.4 x 10^–6^ | KASPar | |  |
| *APOE* | rs429358 |  | 19:45,411,941 | T/C | 0.67 | 3.9 x 10^–39^ | smMIP | |  |
| *APOE(EXOC3L2/MARK4)* | rs73036519 |  | 19:45,748,362 | G/C | 0.91 | 2.4 x 10^–5^ | smMIP | |  |
| *MMP9* | rs142450006 |  | 20:44,614,991 | TTTTC/T | 0.84 | 5.3 x 10^–9^ | smMIP | |  |
| *C20orf85* | rs201459901 | rs117420707 (R^2^=0.99) | 20:56,663,846 | C/A | 0.76 | 3.8 x 10^–12^ | KASPar | |  |
| *SYN3/TIMP3* | rs5754227 |  | 22:33,105,817 | T/C | 0.79 | 5.7 x 10^–16^ | smMIP | |  |
| *SLC16A8* | rs8135665 |  | 22:38,476,276 | C/T | 1.14 | 1.4 x 10^–8^ | smMIP | |  |

*Fritsche LG, Igl W, Bailey JNC, Grassmann F, Sengupta S, Bragg-Gresham JL, et al. A large genome-wide association study of age-related macular degeneration highlights contributions of rare and common variants. Nat Genet. 2016;48(2):134–43.

† The SNP database has been updated and some SNP identification numbers numbers has been updated accordingly.

‡ Ten SNPs from the 52 AMD-associated variants genotyped by the smMIP method did not pass quality controls. These variants were then genotyped by KASP genotyping assays.

ABCA1 = ATP binding cassette subfamily A member 1; ACAD10 = acyl-CoA dehydrogenase family member 10; ADAMTS9 = ADAM metallopeptidase with thrombospondin type 1 motif 9; ADAMTS9-AS2 = ADAMTS9 antisense RNA 2; A = Adenine; AMD = age-related macular degeneration; APOE = apolipoprotein E; APOE (EXOC3L2/MARK4) = apolipoprotein E (exocyst complex component 3 like 2/microtubule affinity regulating kinase 4); ARHGAP21 = Rho GTPase activating protein 21; ARMS2/HTRA1 = age-related maculopathy susceptibility 2/HtrA serine peptidase 1; B3GALTL = beta 3-glucosyltransferase; C = cytosine; CES = Coimbra Eye Study; Chr = chromosome; CETP = cholesteryl ester transfer protein; CFH = complement factor H; CFHR1 = complement factor H related 1; CFHR3 = complement factor H related 3; CFI = complement factor I; CNN2 = calponin 2; COL8A1 = collagen type VIII alpha 1 chain; COL4A3 = collagen type IV alpha 3 chain; CTRB2/CTRB1 = chymotrypsinogen B2/chymotrypsinogen B; C2 = complement component 2; C2/CFB/SKIV2L = complement component 2/complement factor B/ski2 like RNA helicase; C3 = complement component 3; C9 = complement component 9; C20orf85 = chromosome 20 open reading frame 85; G = Guanine; GRS = genetic risk score; NRTN/FUT6 = neurturin/fucosyltransferase 6; KASPar = Kompetitive allele-specific polymerase chain reaction; KMT2E/SRPK2 = Lysine Methyltransferase 2E/ Serine/threonine-protein kinase 2; LIPC = lipase C; NPLOC4/TSPAN10 = NPL4 homolog, ubiquitin recognition factor/tetraspanin 10; MIR6130/RORB = microRNA 6130/RAR related orphan receptor b; MMP9 = matrix metallopeptidase 9; OR = odds ratio; PBX2 = PBX homeobox 2; PILRB/PILRA = paired immunoglobin like type 2 receptor beta/paired immunoglobin like type 2 receptor alpha; PRLR/SPEF2 = prolactin receptor/sperm flagellar 2; RAD51B = RAD51 paralog b; RDH5/CD63 = retinol dehydrogenase 5/ CD63 molecule; Rs id = SNP identification number; SLC16A8 = solute carrier family 16 member 8; SNP = single nucleotide polymorphism; smMIP = single-molecule molecular inversion probes; SYN3/TIMP3 = synapsin III/TIMP metallopeptidase inhibitor 3; TGFBR1 = transforming growth factor beta receptor 1; TMEM97/VTN = transmembrane protein 97/vitronectin; T = Thymine; VEGFA = vascular endothelial growth factor A.
