# Supplementary material for: Interaction between genetics and the adherence to the Mediterranean diet: the risk for age-related macular degeneration. Coimbra Eye Study Report 8
Source: Eye Vis (Lond). 2023 Aug 14;10:38. doi: 10.1186/s40662-023-00355-0 (PMC10424352; doi:10.1186/s40662-023-00355-0)
Supplement: Supplementary file 3 — Additional file 3: Table S3. Genetic characteristics of the major risk variants associated with AMD. [file 40662_2023_355_MOESM3_ESM.docx]

**Supplementary Table 3. Genetic characteristics of the major risk variants associated with AMD.**

|  | Cases  (n = 161) | Controls  (n =451) | *P* value |
| --- | --- | --- | --- |
| Genetic risk score, n (%) |  |  | **<0.001** |
| Major common risk variants MAF,  No. of minor alleles/total No. of alleles (%) |  |  |  |
| *ARMS2/HTRA1* rs3750846, C | 60/322 (18.6 %) | 123/902 (13.6%) | 0.110 |
| *CFH* rs570618, T | 113/322 (35.1 %) | 273/902 (30.3 %) | 0.290 |
| *CFH* rs10922109, A | 113/322 (35.1 %) | 395/902 (43.8 %) | **0.007** |
| *C2/CFB/SKIV2L* rs429608, A | 23/322 (7.1 %) | 124/902 (13.7 %) | **0.005** |
| *C3* rs2230199, C | 55/322 (17.1 %) | 164/902 (18.2 %) | 0.740 |
| Bold values represent statistically significant differences between cases and controls with *P* < 0.05, using the Pearson's Chi-squared test (or Fisher´s exact test, when appropriate) for categorical variables and the Mann-Whitney U test for continuous variables. | | | |
| AMD = age-related macular degeneration; ARMS2 = age-related maculopathy susceptibility 2; HTRA1 = HtrA serine peptidase 1; CFH = complement factor H; C2 = complement component 2; CFB = complement factor B; SKIV2L = Ski2 like RNA helicase; C3 = complement component 3; MAF = minor allele frequency; C= cytosine; A = adenine; T = thymine | | | |
|  | | | |
|  | | | |
